# Long-Standing Overt Ventriculomegaly in Adults (LOVA) With Absent Septum Pellucidum and Spontaneous Ventriculostomy: Report of a Rare Case

**DOI:** 10.7759/cureus.52292

**Published:** 2024-01-15

**Authors:** Jichang Seong, Rakhimov Akmal, Kadyrkhodjayeva Nigora

**Affiliations:** 1 School of Medicine, Central Asian University, Tashkent, UZB; 2 Department of Radiology, AKFA Medline University Hospital, Tashkent, UZB; 3 Department of Neurology, AKFA Medline University Hospital, Tashkent, UZB

**Keywords:** ventricular rupture, ventriculomegaly, spontaneous ventriculostomy, macrocephaly, lova, hydrocephalus, absent septum pellucidum

## Abstract

Long-standing overt ventriculomegaly in adults (LOVA) is a type of chronic hydrocephalus with presumable infant onset characterized by macrocephaly and massive ventriculomegaly that causes clinical presentations in later adult life. We report a case of a 20-year-old man who was referred from the ophthalmology department for further investigation of his visual disturbances. MRI of the head revealed massive ventriculomegaly with an Evan's index of 0.44. A careful investigation revealed coexisting aqueductal stenosis, absent septum pellucidum, ventricular rupture, and spontaneous ventriculostomy. The clinical presentations were relatively mild compared to his MRI findings. He was referred to a neurosurgeon for potential surgical interventions after the administration of conservative hyperosmolar drugs and neuroprotective agents.

## Introduction

Long-standing overt ventriculomegaly in adults (LOVA) is a type of chronic hydrocephalus characterized by macrocephaly with massive enlargement of the third and lateral ventricles that is presumably of infant onset with paradoxical mild clinical presentations in adulthood [1]. Due to the poorly understood pathophysiology and several overlapping clinical and radiological diagnostic criteria with other chronic cerebrospinal fluid (CSF) disorders, LOVA frequently remains undiagnosed and maltreated even in the centers of excellence [2].

In the case of severe long-standing hydrocephalus, a partial or complete absence of the septum pellucidum may occur as a result of septal necrosis secondary to elevated intraventricular pressure [3]. Partial or complete absence of septum pellucidum is rarely an isolated finding and is usually associated with other brain malformations with an estimated incidence of two to three cases per 100,000 in the general population [4]. Spontaneous ventriculostomy is another rare condition observed in the case of chronic hydrocephalus, in which the rupture of the thinnest part of the ependymal layer of the ventricle forms a direct circuit to the subarachnoid space, thereby arresting hydrocephalus temporarily or permanently [5,6]. Clinical resolution of symptoms following spontaneous ventriculostomy makes patients not present for further evaluation and therefore, the incidence of spontaneous ventriculostomy is often under-reported [7]. Here we present a rare combination of LOVA, absent septum pellucidum, and spontaneous ventriculostomy in a 20-year-old male patient.

## Case presentation

A 20-year-old man who works as a sheep keeper in Surkhandarya, a rural region of Uzbekistan, presented to AKFA Medline University Hospital's ophthalmology department with a complaint of distance vision impairment for several years, especially in the right eye. He was diagnosed with simple myopic astigmatism with single dystrophic foci on the right macula. Other examination results were within normal ranges. He was then referred to the neurology department for further investigations. Upon arrival at the neurology department, he confessed that together with the distance vision impairment, he had been suffering from a headache that was pulsatile and stabbing in nature. He reported that the headache had been bothering him for two to three years, and the headache had become severe for the past few days. He also stated that the headaches occurred especially when he lifted weights. Previously, he had visited other local hospitals, but there had been no significant improvements in his symptoms. He denied any previous or chronic illnesses such as diabetes mellitus, tuberculosis, viral hepatitis, and HIV infections. He had no allergy history. He stated that he had a traumatic head injury with loss of consciousness back in 2020 but did not fully explain the course and the treatment. His mother stated that she had a normal pregnancy and delivery without any diseases during the pregnancy. His mother also stated that he had been diagnosed with congenital hydrocephalus and from two months to three years of age, he had been under regular supervision by a local neurosurgeon, and routine lateral cervical puncture for CSF drainage had been performed.

Macrocephaly is defined as a head circumference > 98th percentile in adulthood (53.8 cm for males and 52.9 cm for females) [2]. Upon general inspection, macrocephaly was apparent with a head circumference of 61.8 cm. He was conscious and oriented to time, place, and person, but a mild cognitive deficit was noticed under the Mini-Cog test. Neurological examination results showed that pupillary light reflex, accommodation, and convergence of the eyes were normal with the full range of extraocular muscle movements. No nystagmus was observed. His face was symmetrical without any difficulties in phonation and swallowing. His upper and lower extremities had a full range of motion without any pain. Deep tendon reflexes, muscle tone, and muscle strength of upper and lower extremities were within normal range. His sensations were intact throughout his body. He denied having urinary incontinence. Romberg test and heel-to-toe test were positive with mild gait ataxia.

**Figure 1:**
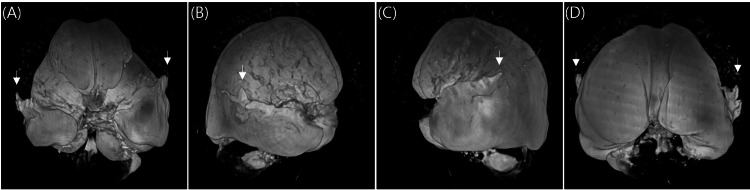
Three-dimensional reconstruction of T2-weighted MRI images of the ventricle. (A) Anterior view. (B) Lateral (right) view. (C) Lateral (left) view. (D) Posterior view. White arrows indicate ventricular ruptures.

**Figure 2:**
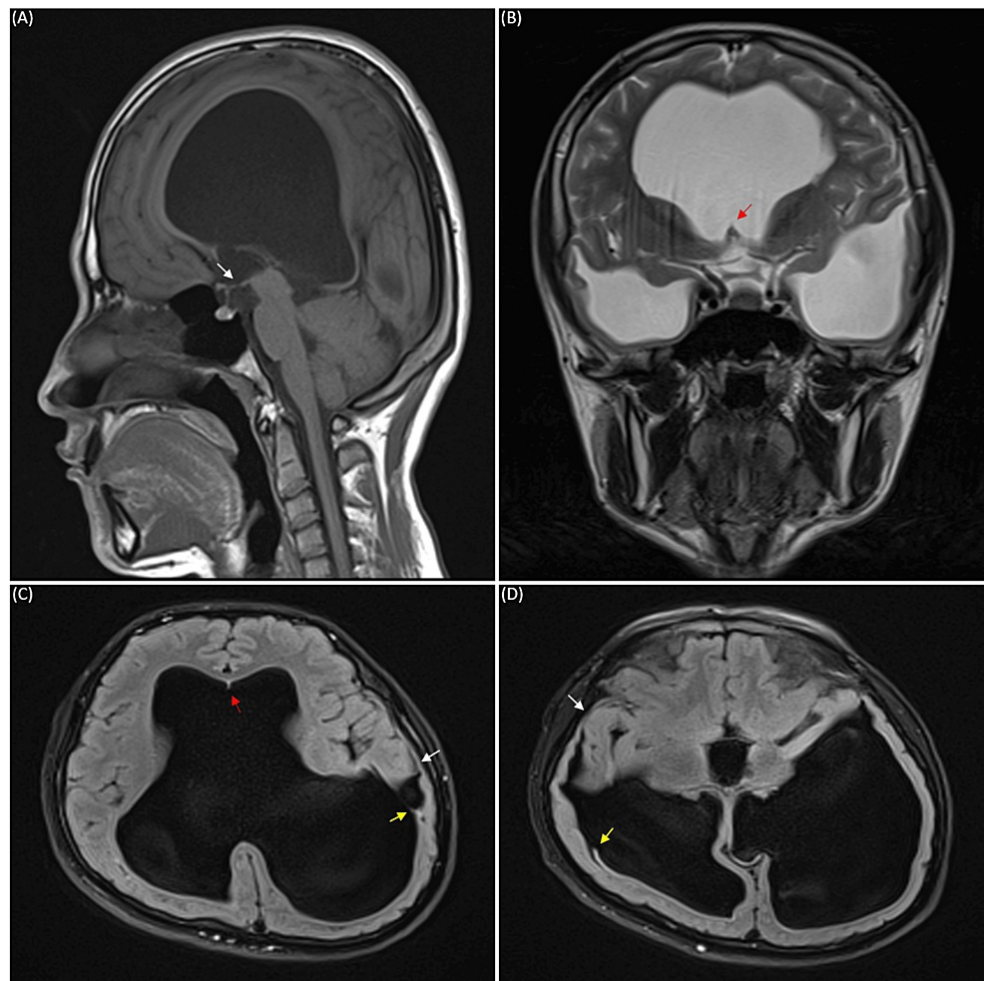
MRI images of the patient’s head. (A) T1-weighted sagittal view. (B) T2-weighted coronal view. (C) and (D) Fluid-attenuated inversion recovery (FLAIR) axial view. White arrows indicate spontaneous ventriculostomy, yellow arrows indicate the rupture of ventricular walls, and red arrows indicate remnants of the septum pellucidum.

**Figure 3:**
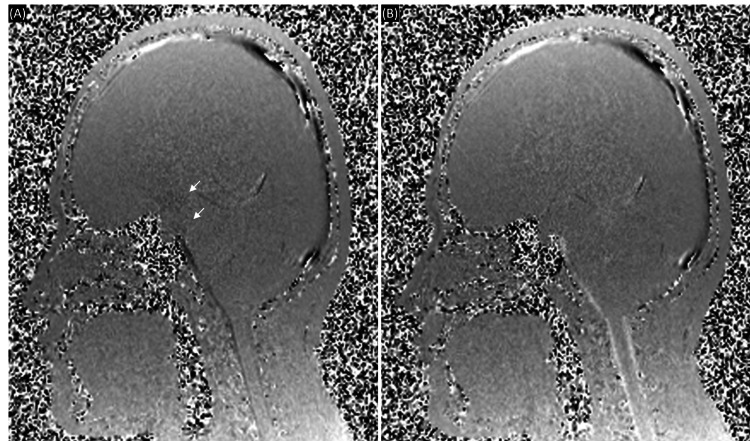
Cine phase-contrast MRI images of the patient's head in sagittal view. The pulsatile flow of cerebrospinal fluid (CSF) is shown in the images. (A) The cranial flow of CSF during diastole is demonstrated in black. (B) The caudal flow of CSF during systole is demonstrated in white. White arrows indicate the flow of CSF through spontaneous third ventriculostomy.

MRI of the head revealed a massive ventriculomegaly. In axial view, the lateral ventricles were dilated to 22 mm on the right anterior horn, 20 mm on the left anterior horn, 56 mm on the right posterior horn, 83 mm on the left posterior horn, 52 mm on the right inferior horn, and 58 mm on the left inferior horn. The third ventricle was expanded to 18 mm and the fourth ventricle remained in normal size and shape. The Evan's index was calculated to be 0.44, indicating ventriculomegaly. Three-dimensional reconstruction of MRI images of the ventricles showed a clear picture of massive ventriculomegaly with atypical wing-like structures that projected to the cortex from the superior aspect of both the right and left inferior horns of the lateral ventricle (Figure 1). Further investigation revealed a thinning of the cortex in the temporal lobes with suspected rupture of the ventricular walls that formed a circuit continuing to the subarachnoid space of the cortex (Figures 2C, 2D). Septum pellucidum was completely absent with small remnants of it seen in between the anterior horns of the lateral ventricle at the level of the genu of the corpus callosum (Figures 2B, 2C). Other characteristic features of hydrocephalus such as thinning of corpus callosum, mild periventricular edema, and effacement of sulci and gyri were also observed. A narrowing at the cerebral aqueduct and compression of the quadrigeminal cistern and cisterna magna were detected. Careful observation revealed a small spontaneous third ventriculostomy that connected the interpeduncular cistern to the floor of the third ventricle (Figure 2A). Cine phase-contrast MRI showed a small CSF flow through the spontaneous third ventriculostomy, but no peak flow dynamics were observed in the cerebral aqueduct (Figure 3). Magnetic resonance (MR) angiogram showed no abnormalities in the anatomy and course of cerebral arteries and venous drainage system, except for the right vertebral artery, which was hypoplastic. Agenesis of the frontal sinus was also observed.

He was diagnosed with LOVA and treated with conservative hyperosmolar drugs and neuroprotective agents. After informing the patient about potential surgical interventions available, he was referred to a neurosurgeon. A follow-up meeting after six months was planned to check the patient’s status.

## Discussion

First described by Oi et al. in 2000, LOVA is a unique form of chronic hydrocephalus with presumable infant onset hydrocephalus that manifests symptoms during adulthood, characterized by macrocephaly measuring more than two standard deviations in head circumference with severe ventriculomegaly and/or evidence of destruction or expansion of sella turcica [8]. It has been proposed that macrocephaly and supratentorial ventricular dilation develop due to the partial or complete obstruction of the cerebral aqueduct prior to the fusion of the cranial fontanelles, and various compensatory mechanisms such as alternative circulation pathways restore the CSF flow so that only in adulthood the symptoms of hydrocephalus develop when the compensatory mechanisms exhaust [2]. Typical symptoms include gait and visual disturbances, urinary incontinence, and cognitive decline [2,8]. Similar to previous studies, we observed symptoms of headache, visual disturbance, gait ataxia, and mild cognitive deficit in our patient. We were also able to observe macrocephaly with severe ventriculomegaly and aqueductal stenosis in our patient but neither destruction nor expansion of sella turcica was present. The overlying pituitary gland was intact with normal size and shape. These may be due to the spontaneous ventriculostomy present in our patient that prevented an increase in intracranial pressure (ICP) and subsequent compression of sella turcica by an expanded third ventricle.

Spontaneous ventriculostomy is a rare condition in which the thinnest part of the ependymal layer of the ventricular wall ruptures and creates a direct circuit to the subarachnoid space, thereby compensating CSF flow in case of obstructive hydrocephalus [5,6]. Previously, spontaneous ventriculostomy was considered an accidental finding during postmortem autopsy, with the reported occurrence at the lamina terminalis of the third ventricle, bilateral posterior wall of the lateral ventricle, and temporal lobe [6]. Recent studies demonstrate that spontaneous ventriculostomy most commonly happens in the third ventricle, with the majority of the instances happening on the floor of the third ventricle followed by lamina terminalis [9]. In cases of severe congenital hydrocephalus with elevated ICP, spontaneous external rupture of the brain through open anterior fontanelle and the leakage of CSF through the skin have been reported [10]. We observed in our patient a spontaneous third ventriculostomy at the floor of the third ventricle and bilateral ventricular rupture at the superior aspect of the temporal horn of the lateral ventricle, which formed a direct circuit through the overlying cortical subarachnoid space. Most cases of spontaneous ventriculostomy happen due to chronic obstructive hydrocephalus, but a case report demonstrated that spontaneous third ventriculostomy occurred in a patient following traumatic brain injury [7]. Thus, both LOVA and past traumatic head injury in our patient may have contributed to the formation of spontaneous ventriculostomy.

Septum pellucidum is composed of two thin translucent membranes that extend from the inferior surface of the corpus callosum to the body of the fornix and separates the anterior horns of the lateral ventricles [3,4]. The function of septum pellucidum has been poorly understood, yet its primary absence has been associated with several anomalies including septo-optic dysplasia, corpus callosum agenesis, and holoprosencephaly. In the case of prolonged severe congenital hydrocephalus, a partial or complete absence of septum pellucidum has been observed and thought to be the result of septal necrosis secondary to increased intraventricular pressure [3]. We observed a complete absence of septum pellucidum in our patient, which may be due to the prolonged obstructive hydrocephalus. The presence of the remnants of the septum pellucidum at the genu of the corpus callosum gives a higher possibility that the absence of septum pellucidum in our patient is an acquired abnormality due to the prolonged obstructive hydrocephalus.

Although surgical interventions do not guarantee the long-term symptomatic relief of LOVA and potential surgical complications are frequent, it is generally accepted that surgical interventions such as endoscopic third ventriculostomy (EVT) and ventriculoperitoneal (VP) shunts are the treatment of choice for the symptomatic LOVA [11]. Several off-label pharmacological interventions are available for treating hydrocephalus, yet their long-term experimental and clinical outcomes remain controversial [12]. It is still a common clinical practice in Uzbekistan to use off-label drugs such as edible glycerol or traditional medicine such as camel’s milk for treating hydrocephalus despite the fact that surgical interventions are free of charge with the appropriate diagnosis and documents. Our patient was left undiagnosed even after several consultations with local doctors and did not know for his entire life that there are possible surgical interventions to treat his condition. In fact, LOVA is frequently unrecognized and untreated even in the centers of excellence around the world [2]. Therefore, continuing education for doctors as well as patients is crucial for early detection and better patient care.

## Conclusions

LOVA is frequently undetected and misdiagnosed in many cases due to its long asymptomatic course and relative rarity. As with any other disease, early detection and interventions are essential in achieving favorable outcomes. To achieve this, continuous education of the doctors as well as patients is crucial. Furthermore, many patients from developing countries face financial difficulties that hinder them from visiting hospitals and receiving appropriate treatments. Governmental healthcare support is crucial in this context.

## References

[1] Tuniz F, Fabbro S, Piccolo D, Vescovi MC, Bagatto D, Cramaro A, Skrap M (2021). Long-standing overt ventriculomegaly in adults (LOVA): diagnostic aspects, CSF dynamics with lumbar infusion test and treatment options in a consecutive series with long-term follow-up. World Neurosurg.

[2] Pirina A, Jusue-Torres I, Desestret V, Albini Riccioli L, Jouanneau E, Palandri G, Manet R (2023). CSF dynamics in long-standing overt ventriculomegaly in adults. Neurosurg Focus.

[3] Siala S, Homen D, Smith B, Guimaraes C (2023). Imaging of the septum pellucidum: normal, variants and pathology. Br J Radiol.

[4] Chun YK, Kim HS, Hong SR, Chi JG (2010). Absence of the septum pellucidum associated with a midline fornical nodule and ventriculomegaly: a report of two cases. J Korean Med Sci.

[5] Rovira A, Capellades J, Grivé E, Poca MA, Pedraza S, Sahuquillo J, Rodríguez-Baeza A (1999). Spontaneous ventriculostomy: report of three cases revealed by flow-sensitive phase-contrast cine MR imaging. AJNR Am J Neuroradiol.

[6] Torkildsen A (1948). Spontaneous rupture of the cerebral ventricles. J Neurosurg.

[7] Abdulsalam HA, Nissiri F, Das S (2018). Letter: spontaneous third ventriculostomy in a patient following traumatic brain injury. Oper Neurosurg (Hagerstown).

[8] Oi S, Shimoda M, Shibata M (2000). Pathophysiology of long-standing overt ventriculomegaly in adults. J Neurosurg.

[9] Aleem Ragab OA, Fathalla H, El Halaby W, Maher W, Hafez M, Zohdi A (2023). Spontaneous third ventriculostomy in cases of aqueductal stenosis: a retrospective case series. World Neurosurg.

[10] Katiyar V, Garg K, Doddamani R, Singh PK, Singh M, Chandra PS (2021). Spontaneous external rupture of hydrocephalus after fontanelle closure: a case report and review of literature. Childs Nerv Syst.

[11] Ved R, Leach P, Patel C (2017). Surgical treatment of long-standing overt ventriculomegaly in adults (LOVA). Acta Neurochir (Wien).

[12] Del Bigio MR, Di Curzio DL (2016). Nonsurgical therapy for hydrocephalus: a comprehensive and critical review. Fluids Barriers CNS.

